# Psychological demands of health professionals in the initial phase of the COVID-19 pandemic

**DOI:** 10.1186/s41155-021-00204-w

**Published:** 2022-01-04

**Authors:** Miryam Cristina Mazieiro Vergueiro da Silva, Bruno Ioschpe, Fernanda Santos Diniz, Graça Maria Ramos de Oliveira, Fabiana Saffi, Amanda Rafaella Abreu Soares, Cristiana Castanho de Almeida Rocca, Antonio de Pádua Serafim

**Affiliations:** Department and Institute of Psychiatry, School of Medicine, University of Sao Paulo, São Paulo, Brazil

**Keywords:** Pandemic, COVID-19, Mental health, Psychological impact, Psychoeducation

## Abstract

Recent literature emphasizes how the specific stressors of the COVID-19 outbreak affect the general population and frontline professionals, including those conducting support or backup activities in health units, which can lead to vicarious traumatization. Vicarious traumatization has been used to describe negative emotional reactions in mental health professionals who assist or treat victims of traumatic events. Thus, the aim of this article is to report the actions used in the face of the psychological demands of professionals in a hospital, who were not on the frontline of COVID-19. We collected their sociodemographic information and checked the psychological impact using the depression, anxiety, and stress scale (DASS-21), which was followed by a psychoeducational program (topics related to COVID-19 control and prevention), and individual psychological care for the most severe cases. A total of 118 professionals participated in this study; they were between 20 and 67 years old. Mild to moderate anxiety was observed in 36.5% of the medical teams, 83.3% of the administrative staff, and 65.7% of general service workers, while stress symptoms were observed in 80.2%, 83.3%, and 59.9%, respectively. Depressive symptoms (35.3%) were more frequent in general service workers. The medical teams reported the benefits of primary psychological care, while the other professionals demanded more institutional support services related to the prevention and use of personal protective equipment. This study highlights relevant psychological demands that have repercussions on the daily lives of professionals. The psychoeducational program was considered positive regarding clarification actions. However, it was not perceived as effective in reducing fear, which may result from vicarious traumatization and requires other intervention modalities.

**Supplementary Information:**

The online version contains supplementary material available at 10.1186/s41155-021-00204-w.

## Introduction

The COVID-19 pandemic has triggered a global crisis in the social, economic, physical, and mental health spheres. Intense psychological reactions, such as anxiety, stress, fear, confusion of thoughts, and increased irritability, have led to difficulties in making coherent decisions (Bao et al., 2020; Chinazzi et al., 2020).

In this scenario, the specific stressors of the COVID-19 outbreak affect the general population and frontline professionals, including those who provide support or conduct indirect activities in health units, and who are sometimes overlooked (Paulino et al., 2021; Serafim et al., 2020). A recent study emphasized that psychological stress, especially indirect trauma caused by the COVID-19 pandemic, should not be ignored, for when stress levels were compared between frontline nurses and nurses from other health units, the latter presented higher levels of vicarious traumatization (Li et al., 2020).

The term “vicarious traumatization” was first established by McCann and Pearlman (1990) to describe the phenomenon in which a specialist psychologist was affected by the trauma transmitted by a person living with a psychiatric condition during or after the psychological consultation process. Currently, the term is used to describe mental health workers who assist or treat victims of traumatic events (Collins & Long, 2003). As it is similar to post-traumatic stress disorder (PTSD), it is necessary to investigate the traits related to emotional vulnerability and psychological reactions, as well as the cognitive aspects affected. People with vicarious traumatization are often witnesses to the suffering of others; that is, the stress is relatively indirect. Preventing it at the organizational level is of paramount importance since exposure to trauma through the provision of care to people who are directly linked to high-risk diseases can result in the development of adverse psychological responses (Al-Mateen et al., 2015).

A study with 339 volunteers about their professional practices and experiences during the pandemic showed that, on average, therapists experienced moderate levels of vicarious trauma, whereas about 15% experienced high levels. A higher level of vicarious traumatization was associated with younger age, less clinical experience, and negative online treatment (Aafjes-van Doorn et al., 2020).

In a study conducted in Malaysia, the level of vicarious trauma due to COVID-19 was significantly higher among frontline health professionals involved in direct diagnosis, treatment, and care of patients and those who face a high risk of infection. The study further suggested that support from various sources such as colleagues, relatives, the public, and the government can play an essential role in the mental health of health professionals (Norhayati, Yusof, & Azman, 2021).

The COVID-19 pandemic is an unprecedented challenge for society. Supporting the mental health of medical staff and affiliated health care workers is a critical part of the public health response. Frontline professionals are at risk not only of developing the adverse health outcomes related to COVID-19 but also of indirectly experiencing trauma through their work (Adams & Walls, 2020). Research into the psychological effects of infectious disease outbreaks, such as severe acute respiratory syndrome (SARS) and H1N1, shows consistent patterns of reactions, and covers the experiences of staff at work, during the quarantine, and those returning to work after taking a leave of absence (Brooks, Rubin, & Greenberg, 2019; Maunder et al., 2003). Staff challenges include not only the increased workload created due to outbreaks but also a fear of getting infecting themselves and passing it on to their families, working with the latest version of protocols, frequently changing personal protective equipment, caring for patients who are extremely ill and quickly deteriorating, and caring for colleagues who have also fallen ill.

In this context, data from previous pandemics, particularly in the post-quarantine period, suggest that health professionals may develop symptoms of post-traumatic stress disorder, depression, and substance use disorders (Brooks, Rubin, & Greenberg, 2019). Data from preliminary studies from China and Italy during the COVID-19 pandemic provide further evidence; for example, health professionals in China reported depression (50.3%), anxiety (44.6%), and insomnia (34.0%) (Søreide et al., 2020).

In view of the pandemic, there is a background of general concern for the safety of health workers and of people infected with COVID-19. These concerns should be considered as the premise of care programs, with the possibility of health professionals having a space to speak openly about concerns and dangers, both real and imaginary, thus configuring themselves as mechanisms for helping these people get through this delicate phase. These aspects are included in the Mental Health and Psychosocial Support for Staff guidelines (MHPSS, 2020).

In the case of COVID-19, health professionals can express two prevalent reactions, the *fear of falling ill* and the *risk of infecting their families* (Zhang et al. 2020). These reactions can lead to strong feelings of being unable to cope due to overload, the same symptoms as in a burnout, along with performance difficulties within the health units in which they work.

In the face of these circumstances, developing programs for mental health care and psychosocial well-being of professionals in health units, whether they are members of a medical team, administrative staff, or general service workers during the crisis period, is as necessary as physical health care, an aspect that has already been highlighted in recent literature (Everly & Lating, 2017).

Therefore, this article presents an account of the emergency psychological care actions implemented in a hospital unit, in view of the demands of professionals not directly involved in the treatment of patients with COVID-19, but who performed their activities in the hospital throughout the quarantine period, closely monitoring all occurrences resulting from the pandemic, and caring for severe psychiatric patients with acute symptomatology. Although we did not use any specific measures to check for vicarious trauma, we structured the discussion in this context.

This program was prepared according to the recommendations of the international literature for psychological care in crisis situations (Everly & Lating, 2017). The objective of the program was to stabilize acute psychological and/or behavioral reactions and attenuate anxiety, impairment, or dysfunction to help these individuals recover some degree of adaptive functionality (resilience), promoting natural coping and resilience mechanisms, and facilitating access to ongoing support or individual psychological support when necessary.

## Methods

### Participants

This is an experience report describing psychological actions in the face of mental health demands on a medical team (physicians, residents, pharmacists, nurses, and psychologists), administrative staff, and general service workers (cleaning, organization, and security professionals) in different sectors of a psychiatric unit in a public hospital in Brazil, between May and December 2020. All professionals were hired through public tenders, except for the general service workers, who were outsourced. Doctors and nurses worked 12-h shifts, psychologists and pharmacists worked 6-h shifts, and administrative and general service staff had 8-h shifts. Women made up most of the sample, 73 were volunteers (61.8%), 61 were married (51.7%), and they had been civil servants for 16.9 years on average. In the administrative sector, 10 (41.6%) were graduates or postgraduates and, on the medical team, 12 (20.3%) were nursing technicians who had completed high school.

The program served 118 professionals aged between 20 and 67 (mean = 41.3 ± 11.7) with complaints related to anxiety, stress, and depression. Of the participants, 59 (50%) were members of a medical team, 24 (20.3%) were administrative staff, and 35 (29.7%) were general service workers. In the face of the demands presented by the professionals regarding cognitive, emotional, and behavioral issues, we organized a health care program. As it was about the development of emergency care to meet the needs of these professionals, voluntary participation in the study was the inclusion criterion, regardless of the level of stress, anxiety, and depression.

### Instruments

#### Psychological demands questionnaire

An ad hoc questionnaire developed to gather information that could guide the actions of mental health support staff. It was based on the Mental Health and Psychosocial Support for Staff guidelines (MHPSS, 2020). This questionnaire contained sociodemographic questions (i.e., age, gender, marital status, professional area and working hours, education, and length of service in the hospital), general health conditions, and information related to the COVID-19 pandemic. Most questions were answered as “yes” or “no,” and addressed their fear of getting infected; their fear of infecting relatives, friends, and colleagues; whether they had difficulty calming down or relaxing; whether they felt overly worried; whether they had symptoms of panic; whether they shivered, as in the case of high anxiety; whether they had difficulty thinking positively; whether they were having difficulty concentrating; whether they felt discouraged or had depressive symptoms; whether they felt more emotional (they cried easily); whether they had difficulties in taking the initiative or making decisions; whether they were more easily irritated, intolerant, and nervous; whether they felt supported or protected by the institution; and whether they needed more information on infection control and preventing COVID-19. The questionnaire is presented as Supplementary Material A in its Portuguese version.

#### Depression, anxiety, and stress scale—DASS-21 (Vignola & Tucci, 2014)

The internal consistency indices adapted for this sample were 0.88, 0.96, and 0.93, respectively. This scale contains 21 questions, divided into 3 subscales, and uses 4-point Likert scales. Each subscale consists of 7 items, which evaluate symptoms related to depression, anxiety, and stress. The DASS-21 provides 3 scores (1 per subscale), whose total sum is between 0 and 21. The highest scores on each scale corresponded to more negative or severe affective states.

#### Evaluation of the psychoeducational program questionnaire

An additional ad hoc questionnaire was developed to assess the participants’ assessment of the activities implemented with the program after its conclusion. The questionnaire included “yes” or “no” questions, as follows: Did you (1) Consider your participation in the intervention useful? (2) Feel confident as to the information provided? (3) Find the guidelines effective? (4) Manage to improve your self-care in relation to the dangers of getting infected? (5) Feel a decrease in the intensity of fears related to the pandemic? (6) Feel better about the factors that bothered you before the activity? (7) Feel more cared for by the institution after participating in the intervention? (8) Believe that you were able to learn a lifelong lesson with this activity? (9) Would you recommend this activity to a colleague? (10) Have you been able to reassure yourself about the real dangers of the pandemic? The questionnaire is presented as Supplementary Material B in its Portuguese version.

### Procedures

This program included a pre-care evaluation to verify the needs and depression, anxiety, and stress symptoms of the participants. Based on the results of the pre-care evaluation, we conducted a psychoeducational program and individual psychological support groups according to the needs of each case.

In the initial phase, defined as pre-care, we used a questionnaire to verify the needs of the professionals from various sectors of the hospital, followed by the investigation of depression, anxiety, and stress symptoms. In the second phase, based on the results obtained from the questionnaire and scale, two support modalities were organized: psychoeducation and individual psychological support. In the third phase (post-care), 6 months after the beginning of the program, we verified how the participants evaluated the psychoeducational program.

### Psychoeducation

The literature has emphasized the effectiveness of psychoeducation as an intervention model aimed at providing patients with data on the diagnosis, etiology, functioning, best treatment, and prognosis of their disease, as well as to educate them about risk and protective factors, side effects of medication and/or treatment, and the importance of a lifestyle that contemplates the management of the difficulties associated with the disease (Sarkhel et al., 2020).

Psychoeducation groups had a channel to contribute to the stabilization of acute reactions related to the impact on the psyche of the experiences of health professionals in the face of the pandemic. This program was structured and applied by members of the hospital’s psychological team. Thus, the applicability of psychoeducation was adapted as an intervention tool in the face of the psychological impact resulting from the COVID-19 pandemic for professionals in a psychiatric hospital, divided into two topics: (a) Perceptions and emotions when facing the pandemic: sessions aimed at reducing and managing fear and guilt about infecting themselves, relatives, colleagues, and patients; stress, anxiety, and fear of having severe symptoms and/or death; feelings of irritability, aggressiveness, excessive worry and sleep, mental fatigue; fatigue about the new hygiene practices; and suggestibility (feeling as if they were infected). (b) Information about COVID-19, official positions (scientific parameters), and impacts on the environment and labor relations, aiming to reduce distortions on contagion; contradictory information from government officials; availability of personal protective equipment; feeling of lack of acceptance, support, and humanization from the institution; recognition of the importance of cleaning and support professionals; and better sanitation, social distancing, and other measures.

Depending on the different working hours and shifts, the psychoeducational program was structured into 12 sessions of 50 min each, twice a month (for morning and evening workers) between May and December 2020. Each session had up to 10 participants per group, in a room with enough space to maintain a clearance of 2 m between each participant, with all of them wearing masks. Each professional participated in an average of eight sessions.

### Individual interventions

In addition to psychoeducational sessions, individual psychological support was required in view of the identification of more pronounced psychological aspects, in participants who needed support in this intervention format. Thus, for each professional, six sessions of brief focal psychotherapy with a maximum duration of 60 min (respecting the safety protocols) were carried out, which could be extended according to the needs of each case. This service was carried out by psychology service professionals who worked in the occupational health area, not part of the study team.

After applying the psychological demands questionnaire and the DASS-21, we observed that 14 (11.8%) of the professionals needed individual psychological support. Seven months after the implementation of the program, six professionals needed to continue with their individual psychological support sessions, in addition to psychiatric contributions due to a previous diagnosis of anxiety and depressive disorder.

### Ethical aspects

Although not a methodologically structured project, we collected the written authorization of the participants who gave their permission for the intervention data to be published while maintaining their anonymity. We registered this study with the Ethics Committee of the Hospital, CAAE (Presentation Certificate for Ethical Appreciation) number 35374920.0.0000.0068.

### Data analysis

Based on the procedures performed, we used the Statistical Package for Social Sciences Software (SPSS 23.0) for data analysis, dividing the data into categorical variables, which were described by frequency and percentage; the continuous variables were described by means and standard deviations. The chi-square test was used to verify differences in frequency proportionality in relation to the variables related to depression, anxiety, and stress levels, with a significance level of *p* < 0.05. To verify how the professionals evaluated the psychoeducational activity, we used a group model.

## Results

The data presented in Table 1 detail the information collected through the pre-care evaluation, with 118 participants, aged between 20 and 67 (41.3 ± 11.7). Fifty-nine professionals worked in the health care sector/medical team (nurses, technicians and assistants, pharmacists and technicians, psychologists, and residents), 35 were responsible for cleaning and organizing the hospital environment, and 24 were responsible for administration. Regarding education, 30.5% had completed elementary school, while 25.4% had completed higher education. Most health professionals were male (72.9%). It was evidenced that more than 57.6% had one to two dependents, and 53.4% did not constitute a risk group.

**Table 1:** Sociodemographic characterization of the sample (*N* = 118 professionals)

	Frequency	(%)
**Department**		
Members of the medical team	59	(50.0)
Administrative staff	24	(20.3)
General service workers	35	(29.7)
**Age**		
20 to 67 years old		
**Educational level**		
Complete elementary school	5	(4.2)
Incomplete elementary school	7	(5.9)
Complete high school	36	(30.5)
Incomplete high school	4	(3.4)
Complete higher education	30	(25.4)
Incomplete higher education	11	(9.3)
Post-graduation	10	(8.5)
Master’s degree	2	(1.7)

Table 2 shows the results of the DASS-21 scale regarding the presence of depression, anxiety, and stress symptoms in the 118 participants.

**Table 2:** Frequency of anxiety, depression, and stress symptoms in the 118 participants

	Med.	Adm	GS	***χ***^**2**^ (***df***)	***p*** value
	***F*** (%)	***F*** (%)	***F*** (%)		
**Depression (DASS-21)**					
Asymptomatic	44 (83.9)	21 (87.5)	22 (62.9)		
Mild	09 (16.1)	03 (12.5)	08 (22.9)	21.1 (4)	0.36
Moderate	**-**	**-**	04 (11.4)		
Severe	**-**	**-**	01 (2.8)		
**Anxiety (DASS-21)**					
Asymptomatic	33 (55.9)	10 (41.7)	10 (28.6)		
Mild	17 (28.8)	11 (45.8)	18 (51.4)	88.6 (4)	0.55
Moderate	07 (11.9)	02 (8.3)	05 (14.3)		
Severe	02 (3,4)	01 (4.2)	02 (5.7)		
**Stress (DASS-21)**					
Asymptomatic	12 (20.3)	04 (16.7)	14 (40.0)		
Mild	21 (35.6)	08 (33.3)	15 (42.9)	1.32 (4)	0.05^a^
Moderate	26 (44.1)	12 (50.0)	06 (17.1)		

*Note: Med.* members of the medical team, *Adm* administrative staff, *GS* general service workers, *DASS-21* depression, anxiety, and stress scale

*F* frequency, *χ*^2^ Chi-square, *df* degree of freedom, *p* value (Chi-square test)

^a^Significant at 95%

In Table 2, it is possible to observe that the sample in general presented a low frequency for depressive symptoms, not showing significant differences (*χ*^2^ (4, *N = 25*) = 21.1, *p* = 0.36). On the other hand, mild anxiety symptoms were more prevalent for the three categories: members of a medical team (28.8%), administrative staff (45.8%), and general service workers (51.4.9%), with no significant differences between these groups of professionals (*χ*^2^ (4, *N = 65*) = 88.6, *p* = 0.55). As for stress symptoms, it was observed that mild symptoms were more common in general service workers. Meanwhile, moderate symptoms were more common in the medical team and administrative staff, with a significant difference (*χ*^2^ (4, *N = 88*) = 1.32, *p* = 0.05).

Based on the information collected through the questionnaire on psychological demands*,* elaborated according to the MHPSS guidelines (IASC, 2007), we organized the data of the 118 participants into 5 groups: (1) vulnerability situation (fear of getting infected *or* infecting a relative or friend); (2) visibility/organizational support; (3) access to information (prevention and care); (4) psychological reactions: difficulties in calming down, feeling panic, difficulties in thinking positively, fear of losing control, shivering, discouragement, crying easily, and irritability; and (5) cognitive aspects: difficulties in making decisions and concentrating. The analysis of these groups was chosen to produce synthesized results. Thus, we grouped the questions by content and analyzed their frequency and percentages.

Table 3 presents the relative percentages for the grouped items. According to the data characterized by group 1, 69 (58.5%) participants were afraid of getting infected. On the other hand, the *fear of infecting relatives and friends* showed more relevance, with a total of 92 (78%) people among the respondents: 49 (41.5%) members of the medical team, 19 (16.1%) professionals of administrative follow-up, and 24 (20.3%) general service workers (group 2, criteria related to organizational support). Only 39 (33.1%) professionals reported that they were supported by the institution, with members of the medical team constituting most of the sample, representing 18.6% of total participants (group 3, access to information to avoid getting infected and prevent the spread of the virus). Given the importance of these characteristics among the professionals working in this area, 83.1% of the interviewees did not need any further information to avoid getting infected.

**Table 3:** Group analysis of program demands—pre-care

		Med.	Adm	GS	Total
		59 (50%)	24 (20.3%)	35 (29.7%)	*N* = 118
		***n*** (%)	***n*** (%)	***n*** (%)	***n*** (%)
**Group 1—vulnerability situation**					
Fear of getting infected	Yes	36 (30.5)	12 (10.2)	21 (30.4)	**69 (58.5)**
	No	23 (19.5)	12 (10.2)	14 (28.6)	49 (41.5)
Fear of infecting a relative or friend	Yes	49 (41.5)	19 (16.1)	24 (20.3)	**92 (78)**
	No	10 (8.5)	5 (4.2)	11 (9.3)	26 (22)
**Group 2—organizational support**					
The person felt was supported by the institution	Yes	22 (18.6)	10 (8.5)	7 (5.9)	39 (33.1)
	**No**	**37 (31.4)**	**14 (11.9)**	**28 (29.7)**	**79 (66.9)**
**Group 3 (access to information)**					
Need for further information to avoid getting infected	Yes	10 (8.5)	5 (4.2)	5 (4.2)	20 (16.9)
	**No**	**49 (41.5)**	**19 (16.1)**	**30 (25.4)**	**98 (83.1)**
Need for further information for prevention	Yes	3 (2.5)	5 (4.2)	3 (2.5)	11 (9.3)
	No	56 (47.5)	19 (16.1)	32 (27.1)	107 (90.7)
**Group 4—psychological reactions**					
Difficulties in calming down	Yes	20 (16.9)	3 (2.5)	2 (1.7)	25 (21.2)
	**No**	**39 (33.1)**	**21 (17.8)**	**33 (28)**	**93 (78.8)**
Feeling panic	Yes	5 (4.2)	**-**	**-**	5 (4.2)
	**No**	**54 (45.8)**	**24(20.3)**	**35 (29.7)**	**113 (95.8)**
Difficulties in thinking positive	Yes	6 (5.1)	2(1.7)	2 (1.7)	10 (8.5)
	**No**	**53 (44.9)**	**22 (18.6)**	**33 (28)**	**108 (91.5)**
Fear of losing control	Yes	8 (6.8)	1 (0.8)	**-**	9 (7.6)
	**No**	**51 (43.2)**	**23 (19.5)**	**35 (29.7)**	**109 (92.4)**
Shivering	Yes	4 (3.4)	**-**	**-**	4 (3.4)
	**No**	**55 (46.6)**	**24 (20.3)**	**35 (29.7)**	**114 (96.6)**
Depressed or discouraged	Yes	7 (5.9)	**-**	2 (22.2)	9 (7.6)
	**No**	**52 (44.1)**	**24 (20.3)**	**33 (30.3)**	**109 (92.4)**
Feeling emotional and crying easily	Yes	11 (9.3)	6 (5.1)	2 (1.7)	19 (16.1)
	**No**	**48 (40.7)**	**18 (15.3)**	**33 (28)**	**99 (83.9)**
Angry/introspective or nervous	Yes	13 (11)	3 (2.5)	4 (3.4)	20 (16.9)
	**No**	**46 (39)**	**21 (17.8)**	**31 (26.3)**	**98 (83.1)**
**Group 5—cognitive aspects**					
Difficulties in making decisions	Yes	7 (5.9)	1 (0.8)	1 (0.8)	9 (7.6)
	**No**	**52 (44.1)**	**23 (19.5)**	**34 (28.8)**	**109 (92.4)**
Difficulties in concentrating	Yes	19 (16.1)	4 (3.4)	2 (1.7)	25 (21.2)
	**No**	**40 (33.9)**	**20 (16.9)**	**33 (28)**	**93 (78.8)**

*Note: Med* members of medical team, *Adm* administrative staff, *GS* general service workers

Based on evidence that demonstrated the psychological impact of the pandemic on society, we investigated the presence of psychological reactions among the interviewees. No relevant answers about psychological reactions were found in this sample. Most interviewees did not identify such characteristics in their professional routine. In relation to the cognitive aspects (group 5), they were also not relevant. However, difficulty in concentrating was still present in 21.2% of the total sample.

In this scenario, it was perceived that the characteristics of the COVID-19 pandemic caused a generalized climate of caution, fear, and uncertainty, especially among members of the medical team, due to several causes, such as the rapid spread of COVID-19 and the severity of the symptoms, ignorance regarding the disease, and deaths among health professionals, as well as the need for adaptation.

In the same line, one should consider the organizational factors that became the heart of the elaboration of this program, such as feelings of not receiving adequate support, concerns about their own health, fear of infecting relatives or other people, difficulties in concentration, and work overload, especially among health professionals.

Table 4 represents the post-intervention data divided into groups: group 1 for organizational factors and group 2 for physical and psychological factors identified after the intervention. Among the employees, 53.4% considered it important to participate in the psychoeducational process, 51.7% felt confident about the information provided and its effectiveness during their orientation, 58.5% would recommend replication of the activity, and 64.4% did not feel satisfied with the institution.

**Table 4:** Evaluation of the program by the 118 professionals—post-care

		Med.	Adm	GS	Total
		59 (50%)	24 (20.3%)	35 (29.7%)	*N* = 118
		***n*** (%)	***n*** (%)	***n*** (%)	***n*** (%)
**Group 1—organizational factors**					
Importance to participating	Yes	26 (22)	14 (11.9)	**23 (19.5)**	63 (53.4)
	No	**33 (28)**	10 (8.5)	12 (10.2)	55 (46.6)
Confidence as to the information provided	Yes	26 (22)	13 (11)	**22 (18.6)**	61 (51.7)
	No	**33 (28)**	11 (9.3)	13 (11)	57 (48.3)
Effectiveness in orientation	Yes	26 (22)	14 (11.9)	**21 (17.8)**	61 (51.7)
	No	**33 (28)**	10 (8.5)	14 (11.9)	57 (48.3)
Institutional support	Yes	19 (16.1)	10 (8.5)	13 (11)	42 (35.6)
	No	**40 (33.9)**	14 (11.9)	**22 (18.6)**	**76 (64.4)**
Activity recommendation	Yes	32 (27.1)	15 (12.7)	22 (18.6)	69 (58.5)
	No	27 (22.9)	9 (7.6)	13 (11)	49 (41.5)
**Group 2—psychological factors**					
The person felt more relaxed	Yes	13 (11)	10 (8.5)	**19 (16.1)**	42 (35.6)
	No	**46 (39)**	14 (11.9)	16 (13.6)	**76 (64.4)**
Decrease in the intensity of fear	Yes	8 (6.8)	10 (8.5)	14 (11.9)	32 (27.1)
	No	**51 (43.2)**	14 (11.9)	**21 (17.8)**	**86 (72.9)**

*Note*: *Med* members of medical team, *Adm* administrative staff, *GS* general service workers)

Regarding the evaluation of the program (Table 4), more than half of the 118 participants considered it important to participate in the activity, felt confident in the information provided, understood which guidelines were effective, and gave their feedback on whether they would recommend these activities to other professionals. Concerning the physical and psychological factors, when we evaluated whether the participants felt more relaxed and had reduced their fear (getting infected, death of a relative, for example), only a third identified this change. This result suggests that psychological and physical symptoms were more intense at that time and could not be relieved with psychoeducation only.

## Discussion

The pandemic has profoundly altered social and work environments in many ways. Social distancing policies, mandatory restrictions, periods of isolation, and anxiety, together with the suspension of productive activities, loss of income, and fear of the future, jointly influenced the mental health of citizens and workers (Zhang et al., 2020; Santos, 2020; Galea, Merchant, & Lurie, 2020; Serafim et al., 2021).

In this article, we reported on emergency psychological actions during the early phase of the COVID-19 pandemic in a public mental health hospital in Brazil. This program lasted for 7 months (between May and December 2020), being structured considering the demands of various groups regarding requests to take a leave of absence and/or psychological support due to anxiety, fear, difficulties in concentrating on work, stress, and concerns about infecting themselves and their relatives, even though they were not acting directly on the frontline of the COVID-19 response. The medical team (physicians, residents, pharmacists, nurses, and psychologists), administrative staff, and general service workers (cleaning, organization, and security) participated in the clinical interventions.

Faced with the uncertainties of the COVID-19 pandemic period, combined with the growing volume of infected and dead people, including health professionals, the data analyzed here showed that most professionals, regardless of their area of activity, experienced mild to severe anxiety symptoms. Regarding stress, the professionals related to clinical activities—that is, those involved in patient care—although not directly related to COVID-19, had moderate stress symptoms, followed by general service workers, who mostly did cleaning work. These results show that even though these professionals were not directly on the frontline, this did not stop them from feeling psychological pressure. Although we had no previous data on the mental health of this population, one finding stood out: the presence of these symptoms culminated in an increase in the demand for psychological care, which led us to associate this demand with the pandemic. By then, we had already found data in the literature corroborating these observations (Zhang et al., 2020; Xiang et al., 2020; Wang et al., 2020).

In this context, a possible way to understand the stress response observed here, even though we did not use measuring instruments for this variable, would be the condition of vicarious traumatization, initially referred to by psychotherapists (McCann & Pearlman, 1990; Aafjes-van Doorn et al., 2020; Al-Mateen et al., 2015), and more recently as a response to addressing the COVID-19 pandemic (Li et al., 2020). However, the understanding of vicarious traumatization goes beyond the therapeutic process and is part of the scope of major disasters, problems, and emergency situations, in which the degree of damage exceeds psychological and emotional tolerances, indirectly causing several psychological abnormalities (Patel-Kerai et al., 2017; Serafim et al., 2020; Sinclair & Hamill, 2007; Zhang et al., 2020).

We highlight the study by Li et al. (2020), in which the characteristics of vicarious traumatization were verified in 214 people from the general population and 526 nurses (234 on the COVID-19 frontline and 292 not on the frontline). The results showed that vicarious traumatization scores for frontline nurses, including scores for physiological and psychological responses, such as anxiety and stress, were significantly lower than those for nurses who were not on the frontline. In addition, they observed that the scores of the general population were also significantly higher than frontline nurses. Li et al. (2020) also emphasized that, although vicarious traumatization is associated with a direct relationship between the professional and the victim, psychological stress should not be ignored, especially the vicarious traumatization caused by the COVID-19 pandemic.

When suffering from vicarious trauma, the professional may present a loss of appetite, fatigue, physical decline, sleep disorders, irritability, a lack of attention, numbness, fear, and despair (Al-Mateen et al., 2015; Creighton et al., 2018). Direct or indirect exposure to the phenomenon triggers chemical and electrical alerts in the brain, preparing the body to cope with tension and regain its internal balance. Thus, tension caused by this physiological preparation to “fight or flee,” mainly through the cortisol hormone, is important for the maintenance of life (Margis et al., 2003). However, excessive exposure to stressors can make this alert unregulated and sensitive to minor environmental changes, such as in the case of the pandemic, which has required constant adaptation and uncertainties; this can deregulate the homeostatic system in cases of anxiety and stress (Graeff & Zangrossi, 2010; Kyrou & Tsigos, 2009).

Considering the results of the evaluation of the psychoeducational program in this context, more than 70% of the participants reported that there was no reduction in fear (of getting infected or the death of a relative, for example). On the one hand, this result shows that the emotional panorama of the participants was significant, which suggests the need for more specific and direct actions such as the psychological first aid (PFA), indicated in emergencies (World Health Organization, 2011). The PFA focuses on education about traumatic stress and active listening, since the person can lose control of their physical and psychological reactions to the situation and experience high levels of stress and anxiety outside the usual patterns (Brooks, Rubin, & Greenberg, 2019). Moreover, since we do not have this objective measure, there may be an association between the intensity of symptoms and vicarious traumatization.

The purpose of the program reported in this study was to apply a set of actions to identify the psychological panorama of hospital professionals and propose actions that could produce an initial welcoming environment, disseminate information that can corroborate the level of knowledge about the severity of the situation, improve self-care behavior, and support those with greater difficulties at the individual level, considering that the literature emphasizes the relevance of identifying and providing intervention for vicarious traumatization at an early stage (Patel-Kerai et al., 2017; Xiang et al., 2020).

In this context, we emphasize that we developed a program during an emergency and our methodology was in line with the precepts of the literature. However, if these interventions were utilized in complex situations such as disasters or earthquakes, the complaints and needs of each affected population group would need to be known (Shanafelt & Noseworthy, 2017). Thus, on the one hand, the program was able to produce relevant information about aspects of the mental health of professionals, and it was perceived as valid, although it was not particularly effective at reducing the psychological symptoms of fear.

A review by Schnitzbauer et al. (2020) presented in its results the importance of developing individual protection standards for employees working in clinics in which there was an initial contact with patients with positive or inconclusive COVID-19 status. Thus, the reports on the *fear of infecting relatives or friends* and the need to receive information to avoid getting infected can be justified through the finding that, in addition to health professionals being a risk group, SARS-CoV-2/COVID-19 has numerous atypical clinical manifestations (Zhang et al., 2020). In addition, there is a set of uncertainties that can corroborate the manifestations of vicarious traumatization, as mentioned above (Li et al., 2020).

Finally, Wang et al. (2020) demonstrated that working on the frontline, receiving insufficient training, and a lack of confidence in protective measures were significantly associated with an increased risk of depression and anxiety. In our research, the group involving psychological reactions did not present significant responses related to symptoms that configured such psychopathological conditions.

This study reported on a psychological care program for professionals working in a hospital environment during the most acute phase of the COVID-19 pandemic, which is considered to have been permeated by important limitations. For example, defining a verification measure for each psychoeducational session certainly provided valuable information for adjusting each session. Having verified the level of vicarious traumatization would also have brought more robust data to this study. In addition, we did not use the DASS-21 in the final evaluation, which prevented us from verifying whether the anxiety, depression, and stress indices changed. However, given the emergency nature of the implementation, the demand for actions that need to be improved in the institutional scope was highlighted, becoming another area linked to outpatient mental health services to support psychology and neuropsychology workers, with this space being important in different institutions in various sectors.

## Conclusion

This study showed that the COVID-19 pandemic is related to fear, confusion, and hopelessness, and had an impact on professionals from different areas, including health. Thus, the results showed relevant psychological demands with repercussions for the daily lives of different professionals. The psychoeducational program was considered positively when it came to clarification actions. However, it was not perceived as effective at reducing feelings of fear, which may result from vicarious traumatization and requires other intervention modalities. That said, we highlighted the need to structure and improve programs that can protect the mental health of professionals who are working to fight the COVID-19 pandemic, regardless of whether they are on the frontline or not.

## Supplementary Information

**Additional file 1.** Supplementary Material A. Questionário de Demandas Psicológicas. Supplementary Material B. Questionário para avaliação do programa de psicoeducação.

## Abbreviations

DASS: Depression, anxiety, and stress scale
PTSD: Post-traumatic stress disorder
SARS: Severe acute respiratory syndrome
MHPSS: Mental health and psychosocial support for staff guidelines
PPE: Personal protective equipment
CAAE: Certificate of presentation for ethical appreciation
SPSS: Statistical package for social sciences software
PFA: Psychological first aid
