# Supplementary material for: Psychological demands of health professionals in the initial phase of the COVID-19 pandemic
Source: Psicol Reflex Crit. 2022 Jan 4;35:2. doi: 10.1186/s41155-021-00204-w (PMC8724226; doi:10.1186/s41155-021-00204-w)
Supplement: Supplementary file 1 — Additional file 1. Supplementary Material A. Questionário de Demandas Psicológicas. Supplementary Material B. Questionário para avaliação do programa de psicoeducação. [file 41155_2021_204_MOESM1_ESM.doc]

Find here the ad hoc questionnaires, including the questions addressing the psychological demands (***supplementary material A***) and the evaluation of the psychoeducational program (***supplementary material B***), in it original version in Brazilian Portuguese. The authors used that to assess the psychological demands and perceptions about the psychoeducation program evaluation. To use this material, please, contact the authors of the manuscript to request permission.

Contact:
Antonio de Pádua Serafim ([a.serafim@hc.fm.usp.br](mailto:a.serafim@hc.fm.usp.br))

Department of Psychiatry, School of Medicine, University of São Paulo, Brazil

**Supplementary Material A. Questionário de Demandas Psicológicas**

| **A – Informações Pessoais** | | | | | |  |
| --- | --- | --- | --- | --- | --- | --- |
| 1. | Idade: __________________________________________________________ | | |  |  |  |
| 2. | Gênero: ________________________________________________________ | | |  |  |  |
| 3. | Escolaridade: ____________________________________________________ | | |  |  |  |
| 4. | Estado Civil: _____________________________________________________ | | |  |  |  |
| 5. | Dependentes Sim ( ) Não ( ) Quantos?__________________________ | | |  |  |  |
| 6. | Quantas pessoas moram com você?__________________________________ | | |  |  |  |
| 7. | Alguém do grupo de risco? Sim ( ) Não ( ) Quem?__________________ | | |  |  |  |
| 8. | Profissão _______________________________________________________ | | |  |  |  |
| 9. | Seu cargo no hospital: ____________________________________________ | | |  |  |  |
| 10. | Jornada de trabalho: _____________________________________________ | | |  |  |  |
| 11. | Há quanto tempo trabalha na área?_________________________________ | | |  |  |  |
|  |  | | |  |  |  |
| **B – Condições de Saúde Geral** | | | | | |  |
| 12. | Você apresenta alguma condição de saúde crônica? | Sim ( ) Não ( ) |  | | | |
| 13. | Faz uso de algum tipo de medicação/tratamento? | Sim ( ) Não ( ) |  | | | |
| 14. | Realiza ou já realizou acompanhamento psiquiátrico? | Sim ( ) Não ( ) | Quanto tempo? | | | |
| 15. | Você apresenta diagnóstico médico atual de algum tipo de transtorno psiquiátrico?  Sim ( ) Não ( ) | | |  |  |  |
| 16. | Faz ou já fez uso regular de algum tipo de medicação psiquiátrica?  Sim ( ) Não ( ) Quanto tempo? | | |  |  |  |
| 17. | Realiza ou já realizou acompanhamento psicoterapêutico?  Sim ( ) Não ( ) Quanto tempo? ____________ | | |  |  |  |
| **C – Reações e Comportamentos Frente a Pandemia** | | | | | |  |
| 18. | Medo de contaminação:  Sim ( ) Não ( ) | | |  |  |  |
| 19. | Medo de contaminar parente, amigo, colega:  Sim ( ) Não ( ) | | |  |  |  |
| 20. | Não se sente amparado ou protegido na instituição:  Sim ( ) Não ( ) | | |  |  |  |
| 21. | Necessidade informações sobre contaminação:  Sim ( ) Não ( ) | | |  |  |  |
| 22. | Necessito de informações sobre prevenção:  Sim ( ) Não ( ) | | |  |  |  |
| 23. | Tenho dificuldade de me acalmar, relaxar:  Sim ( ) Não ( ) | | |  |  |  |
| 24. | Estou com dificuldades para dormir:  Sim ( ) Não ( ) | | |  |  |  |
| 25. | Sensação que estou entrando em pânico:  Sim ( ) Não ( ) | | |  |  |  |
| 26. | Não consigo ter pensamentos positivos:  Sim ( ) Não ( ) | | |  |  |  |
| 27. | Sinto que vou perder o controle:  Sim ( ) Não ( ) | | |  |  |  |
| 28. | Tenho apresentado tremores:  Sim ( ) Não ( ) | | |  |  |  |
| 29. | Tenho apresentado dificuldade para tomar decisões:  Sim ( ) Não ( ) | | |  |  |  |
| 30. | Tenho apresentado dificuldades de concentração:  Sim ( ) Não ( ) | | |  |  |  |
| 31. | Sinto-me desanimado, acho que estou deprimido:  Sim ( ) Não ( ) | | |  |  |  |
| 32. | Sinto-me emotivo, choro com facilidade:  Sim ( ) Não ( ) | | |  |  |  |
| 33. | Sinto-me irritado, intolerante, nervoso:  Sim ( ) Não ( ) | | |  |  |  |
| 34. | Procurei não pensar no Covid-19, mas não consegui:  Sim ( ) Não ( ) | | |  |  |  |
| 35. | Outros informações que queira relatar:­­­­­­­­­­­­­­­­­­­­­­­­­­­_____________________________ | | |  |  |  |

**Supplementary Material B. Questionário para avaliação do programa de psicoeducação**

| **Avaliação de Atividade de Psicoeducação** | |
| --- | --- |
| 1. | Considerou útil a sua participação na intervenção?  Sim ( ) Não ( ) |
| 2. | Sentiu segurança nas informações prestadas?  Sim ( ) Não ( ) |
| 3. | Considerou as orientações eficazes?  Sim ( ) Não ( ) |
| 4. | Conseguiu melhorar o autocuidado em relação aos perigos de contágio?  Sim ( ) Não ( ) |
| 5. | Conseguiu se tranquilizar quanto aos perigos reais da pandemia?  Sim ( ) Não ( ) |
| 6. | Sentiu uma diminuição da intensidade dos medos relacionados à pandemia?  Sim ( ) Não ( ) |
| 7. | Sentiu-se melhor em relação aos fatores que o incomodavam antes da atividade?  Sim ( ) Não ( ) |
| 8. | Sentiu-se mais cuidado pela instituição após participação na intervenção?  Sim ( ) Não ( ) |
| 9. | Você acredita que conseguiu tirar algum aprendizado para vida futura com esta atividade?  Sim ( ) Não ( ) |
| 10. | Você recomenda esta atividade para um colega?  Sim ( ) Não ( ) |
